# Polyethylene glycol/fumed silica composites as shape-stabilized phase change materials with effective thermal energy storage

**DOI:** 10.1039/d2ra08134b

**Published:** 2023-03-08

**Authors:** Giang Tien Nguyen

**Affiliations:** a Faculty of Chemical and Food Technology, Ho Chi Minh City University of Technology and Education (HCMUTE) 1 Vo Van Ngan, Thu Duc Ho Chi Minh City 700000 Vietnam ntgiang@hcmute.edu.vn

## Abstract

Shape-stabilized phase change materials (SSPCMs), adopting polyethylene glycol (PEG) as the phase change material (PCM) confined in fumed silica (FS) as the porous support, and their thermal energy storage properties were thoroughly characterized with varying PEG contents, 60–90 wt%. Given a highly interconnected porous structure and a high porosity (88%), FS offered plenty of cavities to confine a large amount of PEG with interactions such as surface tension, capillary, and interfacial hydrogen bonds (H-bond). The interfacial H-bonds negatively affected the crystallinity of PEG and decreased the thermal energy storage capacity, which could be relieved by a large content of confined PEG. The optimum 80 wt% PEG/FS SSPCM exhibited a high crystallinity of 93.1%, corresponding to a remarkable thermal energy storage capacity of 130.6 J g^−1^, and excellent thermal reliability after experiencing 500 melting/crystallization cycles. Moreover, it exhibited a reduced thermal conductivity compared to pure PEG, promoting heat transfer delay during melting and crystallization processes. The 80 wt% PEG/FS SSPCM combined with gypsum effectively retarded the thermal transfer compared to pristine gypsum, indicating the PEG/FS SSPCMs are suitable for potential applications in building thermal management.

## Abstract 2

Polyethylene glycol/fumed silica shape-stabilized phase change material exhibits effective thermal energy storage, which could be a promising candidate for building thermal energy conversion and management as incorporated with gypsum.

## Graphical abstract

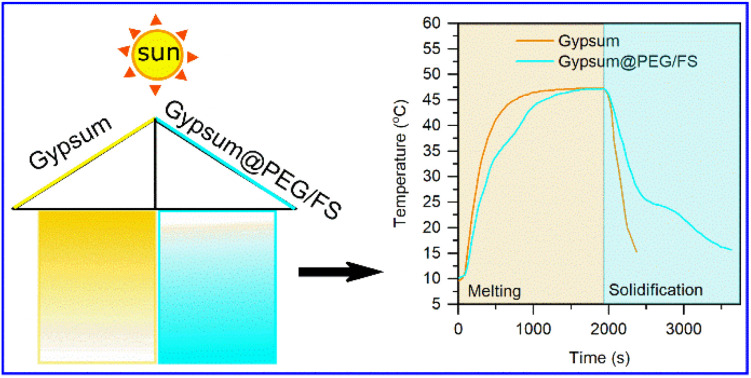

## 1. Introduction

Building thermal management utilizing shape-stabilized phase change materials (SSPCMs) is an advanced technique to achieve comfortable indoor temperatures for human beings and reduce building energy consumption.^1,2^ SSPCMs are composed of phase change materials (PCM) confined in porous supports. The PCMs serve as thermal energy reservoir pools that can store large quantities of latent heat at a defined temperature *via* solid–liquid phase transition. Meanwhile, the porous supports prohibit the leakage of melted PCMs by confining them in nanopores with capillary, surface tension, and interfacial interactions.^3^ Phase transition building thermal management not only has a thermal transfer retardation function but also incorporates storing and releasing thermal energy, narrowing the temperature fluctuation. During the daytime, the PCMs absorb thermal energy and melt; at night, they release the stored thermal energy and crystallize. In practice, SSPCMs are combined with building materials such as gypsum, brick, concrete, insulation panels, and so on.^4,5^

Porous supports play a crucial role in stabilizing PCMs and retaining the PCMs in porous networks after multiple melting/crystallization processes. Pore size and surface properties are key factors determining the crystallization and shape-stability of confined PCMs.^6^ Narrow pores, *e.g.*, micropores, can only retain a limited amount of PCMs and possibly prevent the free movement of PCMs for crystallization, thus reducing the thermal performance. In contrast, large pores, *e.g.*, macropores with micron size, offer wide spaces to infiltrate a large amount of PCM and facilitate the PCM's crystallization, however, molten PCMs are facilely leaked due to weak capillary and surface tension force.^7^ Practically, pore sizes ranging from mesopore to sub-micron size are most suitable to balance the shape-stability and crystallization of confined PCMs.^8,9^ In addition, surface functional groups of porous supports making strong interfacial interactions with PCMs limit the free movements and ordered arrangements of PCMs, negatively affecting the crystallization.^10,11^ Beside stabilizing PCMs, porous supports with a low thermal conductivity can decrease the thermal conductivity of resultant SSPCMs, making them suitable for building thermal energy management. For example, several SSPCMs showed thermal conductivities of 3–3.6 times lower than the pure PCMs as incorporating porous supports having low thermal conductivities including expanded perlite^12^ (0.11 W m^−1^ K^−1^) and silica aerogel^13^ (0.05 W m^−1^ K^−1^). Therefore, it is of vital importance to select porous supports having appropriate porosity and surface properties for achieving SSPCMs with desired thermophysical characteristics.

Frequently used PCMs for building thermal management include polyethylene glycol (PEG), paraffin waxes, fatty acids, and salt hydrates.^1,14^ Of them, PEG shows high thermal attractive thermophysical merits such as high thermal stability, high thermal reliability, and high heat storage capacity of approximately 190 J g^−1^, comparable to fatty acid and salt hydrates.^15,16^ Moreover, PEG exhibits low volume change, non-corrosion, easy availability, and inexpensiveness, which are suitable for large-scale industrial utilization.^16,17^ However, the numerous O atoms and the –OH groups of the PEG chain easily form interfacial hydrogen bond (H-bond) interactions with polar functional groups on porous support surfaces, causing compromised crystallinity, making it fastidious to choose appropriate porous supports. Wang *et al.*^18^ and Feng *et al.*^19^ used mesoporous silica to support PEG, presenting almost 0% crystallinity because of the H-bonds between PEG chains and surface silanol groups. Thus, the obtained thermal energy storage capacities were nearly 0 J g^−1^. A similar phenomenon was reported by Qian *et al.*,^20^ studying a composite of 1-octadecanol impregnated in mesoporous silica. Results showed that the impregnated 1-octadecanol was achieved at low crystallinity of only 28.7% although the mesoporous silica offered sufficient space to stabilize up to 70 wt% 1-octadecanol. Surface modification of porous supports is the most straightforward way to reduce the H-bond interactions and retrieve thermal performance for SSPCMs, however, this strategy often requires expensive reagents and strictly controlled reactions, challenging the scaling-up applications.

Our recent study employed fumed silica (FS) as porous support to confine 1-octadecanol.^21^ FS consisted of nanoscale particles aggregated into a highly interconnected porous structure with combined micro, meso, and macropores, high total pore volume (17 cm^3^ g^−1^), and high porosity (88%). The large pore volume of FS allowed to stabilize up to 75 wt% 1-octadecanol without any leakage owing to the existence of H-bond interactions between the two components, meanwhile, the 1-octadecanol exhibited high crystallinity up to 92.7%. Thus, 1-octadecanol/FS SSPCM was achieved at a high heat storage capacity of 160.3 J g^−1^. These results suggested that the FS could offer a unique porous structure to provide sufficient storage voids as well as transport paths to PCMs. In addition, FS was a cheap and ordinary material, and possessed very low thermal conductivities (0.045 W m^−1^ K^−1^),^22,23^ even lower than the other porous supports (expanded perlite, silica aerogel), thus facilitating large-scale utilization in building thermal energy management. Thus, it could suggest producing efficient and low-cost SSPCMs in which FS can be directly used to confine PCMs without any additional surface modifications. Although the composite of 1-octadecanol confined in FS has been carefully studied, to the best of our knowledge, a lack of investigation on the incorporation of FS and PEG for SSPCM was reported. The long PEG chain with numerous O atoms and two –OH groups at the chain ends would interact differently with FS surfaces compared to the short 1-octadecanol molecule with only one –OH group. It results in a knowledge gap on thermal performance and restricts the insights into the crystallinity of PEG/FS thermal energy storage material.

In light of the above discussion, this work reports a comprehensive investigation of the properties and thermal performance of PEG confined in FS to obtain PEG/FS SSPCMs. A sequence of PEG/FS SSPCMs with increasing PEG mass ratios (60, 70, 80, and 90 wt%) were simply synthesized employing solvent-assisted impregnation method. The PEG/FS SSPCMs were first characterized for morphology, microstructure, chemical compatibility, and leakage resistance. Then, the thermal properties including phase change behaviors, crystallization, thermal stability, and thermal reliability were thoroughly investigated and discussed with the varying PEG contents. The porous structure of FS allowed to impregnation of a very large quantity of PEG (80 wt%) and promoted a high crystallinity (∼93%). In addition, the thermal transfer retardation of PEG/FS SSPCM incorporated into gypsum was also evaluated. The PEG/FS SSPCM added gypsum retarded the heat transfer compared to the pristine gypsum, having high potential to save energy in buildings.

## 2. Materials and experimental methods

### 2.1 Materials

Polyethylene glycol (PEG, molecular weight 1000) was purchased from Shanghai Zhanyun Chemical (China). Fumed silica (FS, Aerosil 200) was purchased from Evonik Degussa (Germany). Absolute ethanol was acquired from Ghtech (China).

### 2.2 Preparation of PEG/FS SSPCMs

FS was dried at 200 °C for 24 hours before usage. PEG/FS SSPCMs were prepared using a solvent-assisted impregnation method.^24^ A calculated amount of PEG was dissolved in ethanol, and then an appropriate quantity of FS was added to the solution. The mixture was stirred at ambient temperature for 2 h to allow the infiltration of PEG into FS porous network. The mixture was then heated at 80 °C for evaporating ethanol until obtaining white powder. Finally, the white powder was dried at 80 °C for 24 h to completely eliminate ethanol, resulting in PEG/FS SSPCMs. Varying PEG contents (60, 70, 80, and 90 wt%) of PEG/FS SSPCMs were prepared.

### 2.3 Characterization methods

The morphology was observed by field-emission scanning electron microscope (FE-SEM, Hitachi S4800, Japan). The porosity was investigated by N_2_ adsorption–desorption isotherm (MicroActive TriStar II Plus, Micrometrics, US). The Brunauer–Emmett–Teller (BET) theory and the non-local density functional theory (NLDFT) were applied to calculate surface area and pore size distribution, respectively. The chemical composition was examined by Fourier-transformed infrared spectroscopy (FTIR 4600, Jasco, Japan) within a wavenumber range of 400–4000 cm^−1^. The crystallization properties were investigated by X-ray diffraction (XRD, Empyrean, Malvern, UK) using Cu Kα radiation, 2*θ* of 5–50°. The phase change behaviors were examined by differential scanning calorimetry (DSC 214 Polyma, Netzsch, US) in a temperature range of −10–65 °C, temperature ramp rate of 5 °C min^−1^, and N_2_ purge gas of 20 mL min^−1^. The thermal stability was studied by thermogravimetric analysis (TGA, Labsys Evo TG-DSC 1600, Setaram, US) in a temperature range of 30–700 °C, temperature ramp rate of 10 °C min^−1^, and N_2_ purge gas of 20 mL min^−1^. The thermal conductivity was investigated by the transient plane source method (TPS 3500, Hot Disk AB, Sweden).

For shape-stability test, the materials were compressed into round blocks (30 mm × 10 mm) and then put on filtered papers and treated in an oven at 60 °C (approximately 20 °C above the melting temperature of PEG) for 60 min. Afterward, the materials were removed from the filter papers and carefully observed to detect the stains of PEG. The shape-stability was further evaluated after 200 repeatedly melting/crystallization cycles. The round block of material was placed in an oven for 30 min at 60 °C (∼20 °C above the melting point of PEG/FS SSPCM) for the melting process. Next, the sample was moved into a refrigerator at 5 °C (∼20 °C below the crystallization point of PEG/FS SSPCM) for the crystallization process. The thermal reliability was tested for 500 melting/crystallization cycles (0 ↔ 60 °C). Approximately 1 g of material in a glass vial was moved back and forth between a low-temperature ice bath (0 °C) and a high-temperature oil bath (60 °C). The dwell time was 4 min at each bath.

**Fig. 1:**
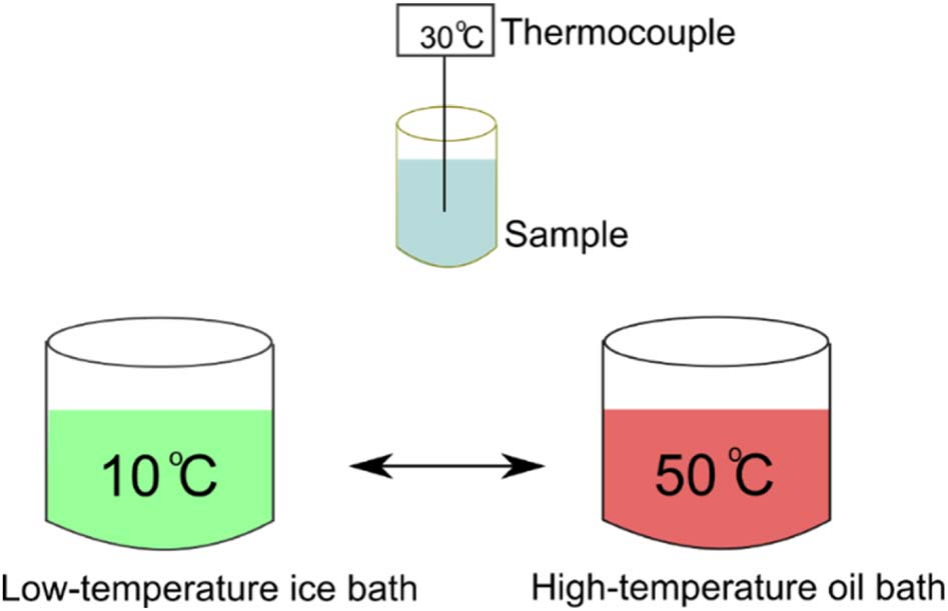
Illustration of apparatus for heat transfer retardation evaluation.

The heat transfer retardation of gypsum and mixtures of gypsum and the 80 wt% PEG/FS SSPCM at 10, 20, and 30 wt% of SSPCM was tested using a homemade apparatus, as illustrated in Fig. 1. Briefly, each material (30 g) was compressed in a cylindrical container (30 mm × 100 mm). The material was initially conditioned at a low-temperature ice bath (10 °C) and then shifted to a high-temperature oil bath (50 °C) for the heat absorption process. When the temperature reached a plateau, the material was shifted back to the low-temperature ice bath for the heat release process. The temperature fluctuation during the test was measured with an Ika ETS-D5 thermocouple.

## 3. Results and discussion

### 3.1 Characterization of PEG/FS SSPCMs

**Fig. 2:**
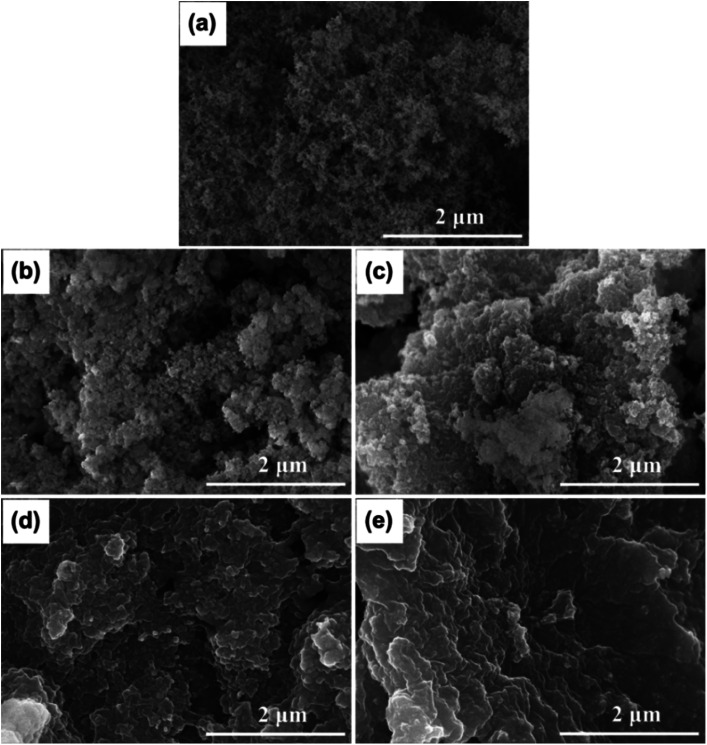
SEM images of (a) FS, (b) 60 wt% PEG/FS, (c) 70 wt% PEG/FS, (d) 80 wt% PEG/FS, and (e) 90 wt% PEG/FS.

**Fig. 3:**
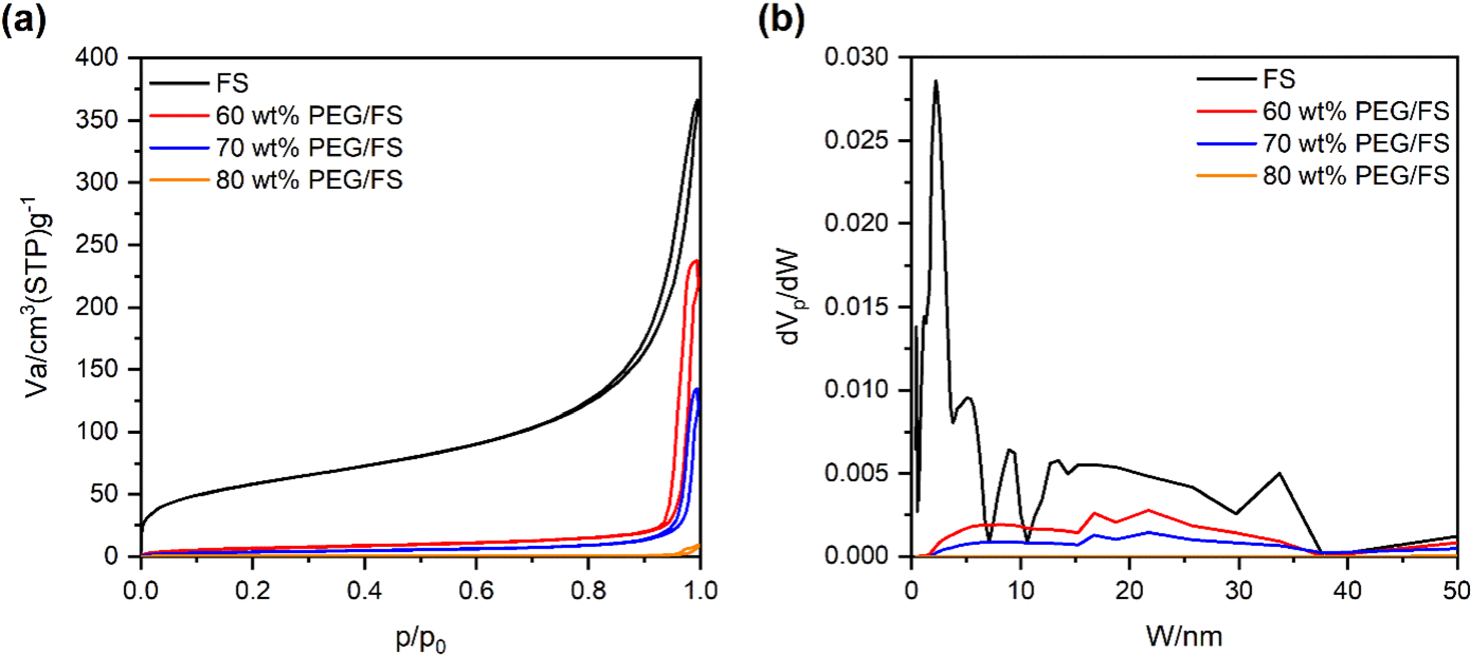
(a) N_2_ adsorption–desorption isotherms of FS and the prepared 60–80 wt% PEG/FS SSPCMs, and (b) the corresponding pore size distribution curves.

The morphology and porosity of the prepared PEG/FS SSPCMs compared to pristine FS were studied by SEM images (Fig. 2) and N_2_ adsorption–desorption isotherms (Fig. 3). The microstructure characters of FS have been carefully reported in our recent study.^21^ In summary, FS included nanoparticles aggregated into a highly interconnected porous scaffold (Fig. 2a) with a large ratio of macropores ranging from 50–150 nm, allowing the impregnation of PEG. The Brunauer–Emmett–Teller (BET) surface area and micro–mesopore volumes of FS were 208 m^2^ g^−1^ and 0.54 cm^3^ g^−1^, respectively, as calculated from the N_2_ adsorption–desorption isotherm (Fig. 3a). Additionally, FS had a great total pore volume of 17 cm^3^ g^−1^ and large porosity of 88%, as obtained from mercury intrusion porosimetry.^21^ When 60–90 wt% of PEG were increasingly impregnated in FS, the FS porous network was gradually filled (Fig. 2b–e), indicating successful impregnation. The N_2_ adsorption–desorption isotherms of the 60–80 wt% PEG/FS SSPCMs exhibited gradually lowered N_2_ adsorption with increasing PEG content (Fig. 3a), which was consistent with the successive disappearance of peaks in the corresponding PSD curves (Fig. 3b), further confirming the successful impregnation. The PDS curve at 60 wt% SSPCM (Fig. 3b) showed a strong peak intensity reduction in the narrower pore region of below 15 nm compared to the wider one of 15–50 nm. The peak intensity in both narrower and wider regions was further reduced with increasing PEG content to 70 wt% and approaching zero at 80 wt%. These results indicated that PEG first infiltrated into the narrower pores and then wider ones during the impregnation process. The FS surfaces and macropores could still be observed for SSPCMs with 60–80 wt% of PEG (Fig. 2b–d). As increasing PEG content to 90 wt% (Fig. 2e), however, the FS surfaces were covered with PEG, suggesting an excessive amount of PEG at this ratio.

**Fig. 4:**
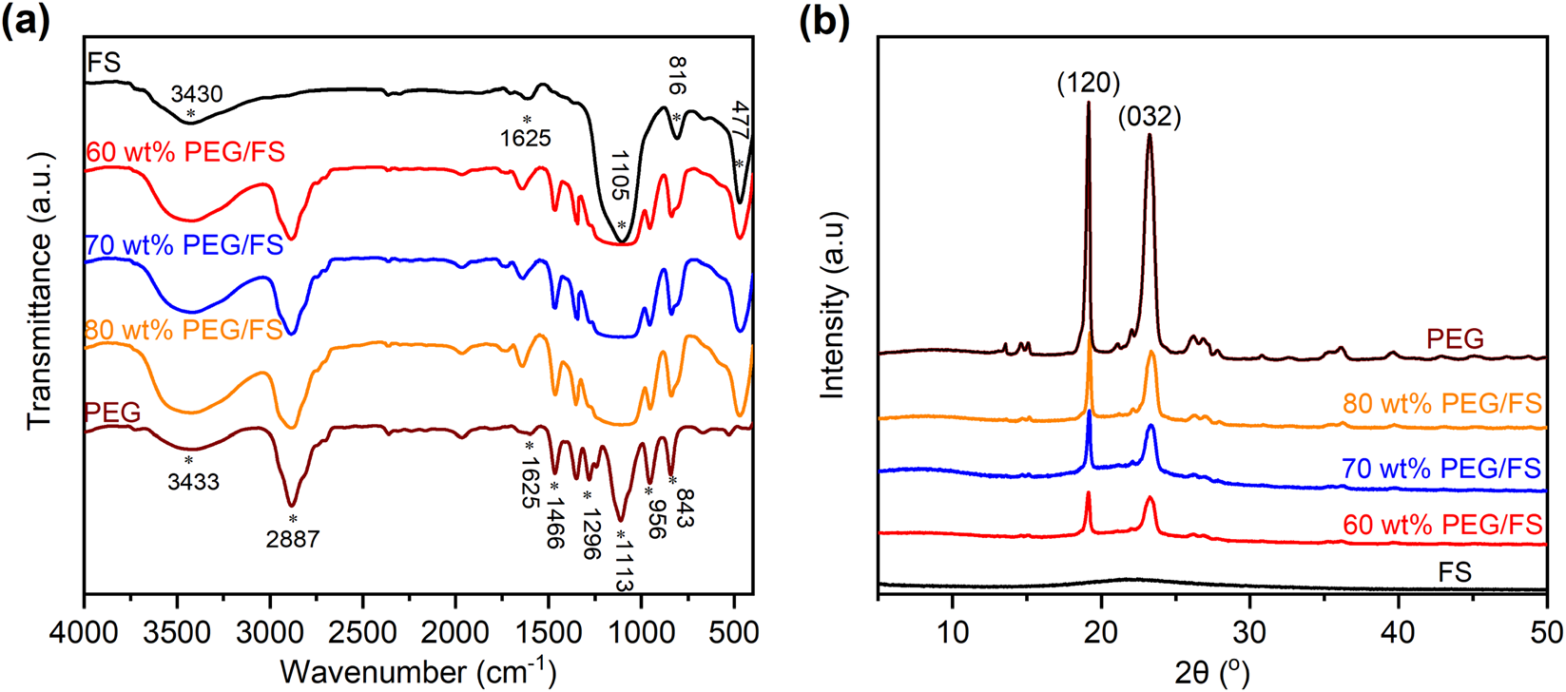
(a) FTIR spectra of FS, PEG, and PEG/FS SSPCMs at 60, 70, and 80 wt% PEG, and (b) XRD patterns of FS, PEG, and PEG/FS SSPCMs at 60, 70, and 80 wt% PEG.

The chemical properties of the prepared PEG/FS SSPCMs were investigated by FTIR and XRD methods. Fig. 4a compares the FTIR spectra of the prepared SSPCMs at 60, 70, and 80 wt% PEG to pure FS and PEG. In the spectrum of PEG, the peaks at 2887, 1466, 1296, 956, and 843 cm^−1^ were assigned to the C–H vibrations, and the peak at 1113 cm^−1^ was assigned to the C–O–C vibration.^17^ The peak at 3433 cm^−1^ was due to the overlapped stretching vibrations of the O–H group from PEG and adsorbed water.^17,25,26^ In the spectrum of FS, the Si–O–Si vibrations were characterized at 1105, 816, and 477 cm^−1^. The silanol groups (Si–O–H) on FS surfaces exhibited a typical vibration at 3430 cm^−1^ which overlapped with the O–H stretching vibration of adsorbed water.^21^ The presence of adsorbed water in pristine PEG and FS could be further observed with bending vibrations at 1625 cm^−1^.^27^ The prepared SSPCMs combined characteristics of the neat materials without any new peaks. It was noted that the adsorbed water still presented in the SSPCMs, characterized by the O–H stretching vibration at approximately 3430 cm^−1^ and O–H bending vibration at 1625 cm^−1^. In the SSPCM, both PEG and FS were hydrophilic, making them unavoidably adsorbing moisture from the air. The adsorbed water amount was computed to be 1–2 wt% by TGA (see later in Fig. 6).

The XRD patterns of the prepared SSPCMs at 60, 70, and 80 wt% PEG (Fig. 4b) showed strong crystal peaks originating from PEG while no crystal peaks from FS appeared because of the amorphous structure of the siliceous material. Indeed, two sharp peaks at 2*θ* of 19.3 and 23.3° were exactly matched to the (120) and (032) planes of PEG crystal, respectively.^28^ These results indicated that PEG and FS were physically compounded in SSPCM without any chemical reactions and the crystallization properties of PEG were maintained after incorporation with FS, even with the presence of adsorbed water.

### 3.2 Phase change properties of PEG/FS SSPCMs

**Fig. 5:**
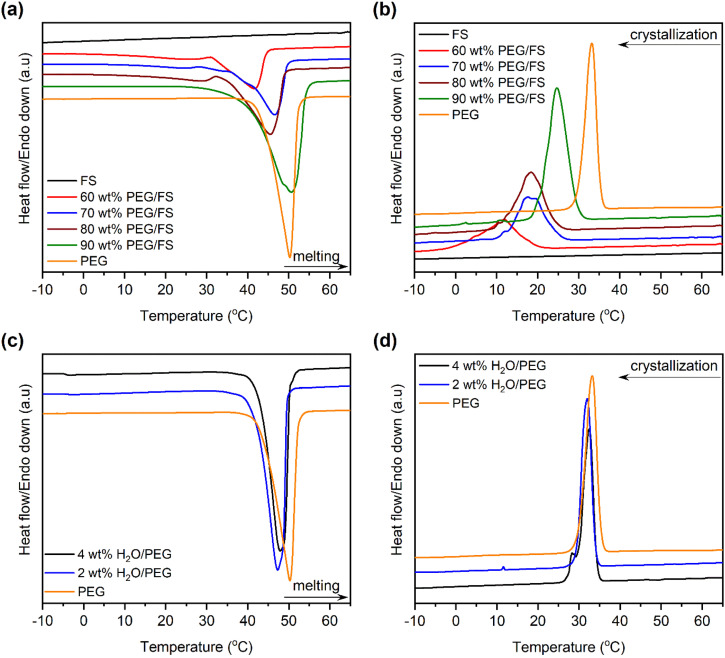
(a) Melting DSC curves of PEG and the prepared SSPCMs, (b) crystallization DSC curves of PEG and the prepared SSPCMs, (c) melting DSC curves of PEG and H_2_O/PEG mixtures, and (d) DSC curves of PEG and H_2_O/PEG mixtures.

The phase change properties were studied by the DSC method, characterizing the melting/crystallization temperature (*T*_M_/*T*_C_) and melting/crystallization enthalpy (Δ*H*_M_/Δ*H*_C_). DSC curves of pure PEG and the prepared PEG/FS SSPCMs were displayed in Fig. 5. The detailed phase change properties derived from the DSC curves were shown in Table 1. The bare FS showed no phase change within the tested temperature. The prepared SSPCMs exhibited suppressed phase change temperatures compared to pure PEG although they all showed a single phase change model in both melting and crystallization. The phase change temperature suppression increased with the decreased PEG content in the SSPCMs. Indeed, while the 90 wt% SSPCM showed *T*_M_ and *T*_C_ of 8.1 and 5.4 °C lower than the pure PEG, the values increased to 12.2 and 15.1 °C as reducing PEG content to 60 wt%, respectively. As confined in the porous network of FS, PEG chains were strained, causing the reduced phase change temperatures.^29^ When PEG was infiltrated into the FS porous structure, it first filled into the narrower pores, *i.e.*, micro–mesopores, and then the wider ones *i.e.*, macropores, as discussed in Section 3.1. PEG chains confined in narrower pores were subjected to more intense strain compared to those in larger pores. Consequently, SSPCMs with lower PEG content showed lower phase change temperatures. Similar results were also found in the literature.^5,29,30^

**Table 1:** Phase change properties of PEG, prepared PEG/FS SSPCMs, and H_2_O/PEG mixtures

Material	*T*_M_ (°C)	*T*_C_ (°C)	Δ*H*_M_ (J g^−1^)	Δ*H*_C_ (J g^−1^)	*F* (%)
PEG	42.6	35.6	175.4	177.8	100.0
60 wt% PEG/FS	30.4	20.5	92.7	96.2	89.1
70 wt% PEG/FS	33.6	26.1	112.5	113.9	91.6
80 wt% PEG/FS	34.4	26.4	130.6	132.4	93.1
90 wt% PEG/FS	34.5	30.2	149.5	151.8	94.8
80 wt% PEG/FS 500 cycles	30.2	26.3	131.2	133.1	93.5
2 wt% H_2_O/PEG	41.0	34.4	172.6	173.2	98.9
4 wt% H_2_O/PEG	41.1	34.5	170.9	170.5	98.6

Another reason is possible that the partial dissolution of PEG in the adsorbed water also induced the phase change temperature suppression. In the SSPCM, water mainly dispersed in PEG because PEG and water could dissolve together while FS could not dissolve water although a thin film of water could be adsorbed on its surfaces. Assuming that all water was dispersed in PEG, the water content in PEG was calculated to be a maximum of 3.5 wt%. To study how PEG behaves when it is partially dissolved in water, mixtures of water and pure PEG (H_2_O/PEG) with water contents of 2 and 4 wt% were prepared and characterized by DSC. The DSC curves of pure PEG compared to those of H_2_O/PEG mixtures are exhibited in Fig. 5c and d, and the detailed phase change properties are shown in Table 1. The partial dissolution of PEG in water did decrease the melting and crystallization phase change temperatures of PEG by approximately 1.5 and 1.0 °C, respectively. For comparison, the melting and crystallization temperature suppression of 60–90 wt% PEG/FS SSPCMs FS were within 8.2–12.2 °C and 5.4–15.1 °C, respectively, much higher than those of H_2_O/PEG mixtures. Our recent reports showed that the phase change temperature of other PCMs (1-octadecanol,^21^ stearic acid,^22^*n*-octadecane^31^) was also remarkably suppressed as confined in FS without the partial dissolution of PCMs in water. These results demonstrated that the confinement effects suppressed the PEG phase change temperatures more strongly than the water dissolution effects. Overall, the phase change temperature suppression of PEG in the SSPCM form was due to both confinement and adsorbed water.

From Table 1, the PEG content could reflect the thermal energy storage capacity of SSPCMs since only PEG owned the ability of thermal energy absorption and release. The pure PEG showed Δ*H*_M_ and Δ*H*_C_ of 175.4 and 177.8 J g^−1^, respectively. The prepared SSPCMs exhibited lower Δ*H*_M_ and Δ*H*_C_ values compared to the pristine PEG, attributed to the presence of FS which reduced the PEG mass ratio compared to the pristine PEG. Moreover, the crystallinity of PEG confined in the FS porous network could be impacted due to confinement and interfacial interaction effects, causing lowered thermal energy storage capacity. The crystallinity of PEG can be effectively evaluated by calculating the crystallization fraction (*F*, (%)) using eqn (1):^32^1
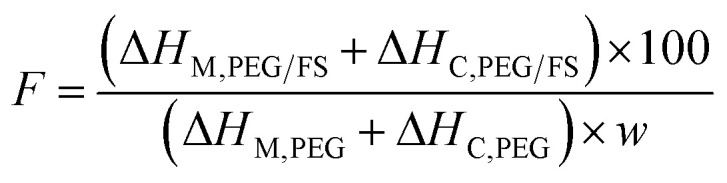
where Δ*H*_M,PEG/FS_ and Δ*H*_C,PEG/FS_ are the melting and crystallization enthalpies of the prepared PEG/FS SSPCMs, respectively. Δ*H*_M,PEG_ and Δ*H*_C,PEG_ are the melting and crystallization enthalpies of pure PEG, respectively. *w* is the mass ratio of PEG in the SSPCMs.

The obtained *F* values (Table 1) were below 100% for all SSPCMs, indicating the crystallinity of PEG was negatively affected in SSPCM form. The *F* values increased with the increasing PEG content, ranging from 89.1–94.8% as increasing PEG content from 60–90 wt%. A possible reason is that the confinement effects and interfacial H-bond interactions lowering the crystallization fraction of PEG could be reduced at high PEG contents. At low PEG content, more PEG proportion resided on FS surfaces, filling in narrower pores, and resulting in more movement restriction of PEG and interfacial H-bonds. Increasing PEG content increased the PEG resided in wider pores and simultaneously decreased the contacting ratio between PEG and FS surfaces, thus increasing the crystallization fraction. In addition, the effects of the partial dissolution of PEG in water on the crystallinity of PEG were also investigated with the H_2_O/PEG mixtures, as shown in Table 1. PEG in the H_2_O/PEG mixtures could exhibit high crystallization fractions (>98.6%), higher than those of PEG/FS SSPCMs (89.1–94.8%), indicating the partial dissolution in water did not greatly affect the PEG crystallinity. Our recent study^21^ showed that the crystallinity of 1-octadecanol was significantly depressed as confined in FS due to confinement effects and interfacial H-bond interactions without water dissolution effects. Therefore, it could be concluded that the crystallinity reduction of confined PEG was mainly due to the confinement effects and H-bond interactions, and slightly due to the adsorbed water.

### 3.3 Thermal stability and shape-stability of PEG/FS SSPCMs

**Fig. 6:**
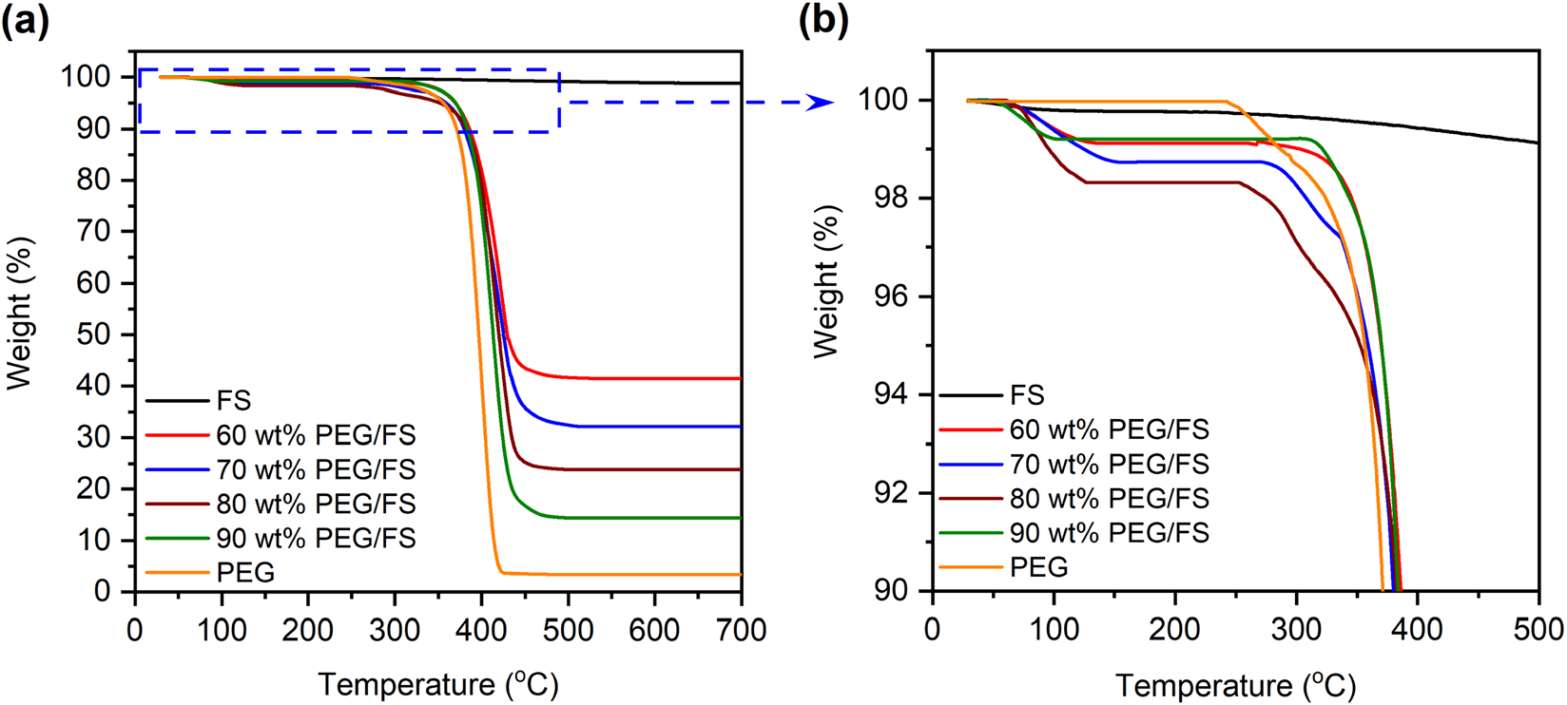
(a) TGA curves of pristine FS, PEG, and the prepared SSPCMs and (b) corresponding TGA curves at higher magnification within the 90–100 wt% range.

Thermogravimetric analysis (TGA) was employed to study the thermal stability of the prepared SSPCMs compared to pure FS and PEG in the range of 30–700 °C, as shown in Fig. 6a. FS exhibited a slight weight loss of 1.2 wt% due to the loss of adsorbed water and surface silanol groups.^30^ The pure PEG exhibited one-step thermal decomposition within a temperature range of 368–426 °C, corresponding to 3.4% residual mass (96.6% PEG effective content). The residue was considered impurities in PEG that did not contribute to the heat adsorption and release process.^16^ The prepared SSPCMs exhibited weight loss in a two-step model. The first step (Fig. 6b) occurred within a temperature range of 80–150 °C accompanied by weight loss of 1–2 wt%, attributing to the loss of adsorbed water. This observation was consistent with FTIR results (Section 3.1) that showed the presence of adsorbed water in the prepared SSPCMs. The second step was due to the composition of PEG occurring within a temperature range of 380–443 °C, slightly higher than the pure PEG. In SSPCM form, PEG chains were restricted in the porous network by capillary, surface tension force, and H-bond interactions,^17,19^ which delayed the spillover of PEG, thus enhancing thermal stability. The thermal decomposition temperature range of the prepared SSPCMs was far higher than the ambient temperature. Thus, PEG/FS SSPCMs possessed good thermal stability for building thermal management applications. In addition, the residual mass of the 60, 70, 80, and 90 wt% PEG/FS SSPCMs was found to be 41.5, 32.1, 23.8, and 14.4 wt%, corresponding to total adsorbed water and PEG effective content of 58.5, 67.9, 76.2, and 85.6 wt%, respectively. After substituting weight loss due to absorbed water and further normalizing using a reported method,^16^ the PEG contents in 60–90 wt% PEG/FS SSPCMs were calculated to be 59.5, 69.2, 77.9, and 87.8 wt%, respectively. These values were highly consistent with the prepared ratios of 60, 70, 80, and 90 wt%, respectively, indicating PEG was uniformly distributed throughout FS porous network.

**Fig. 7:**
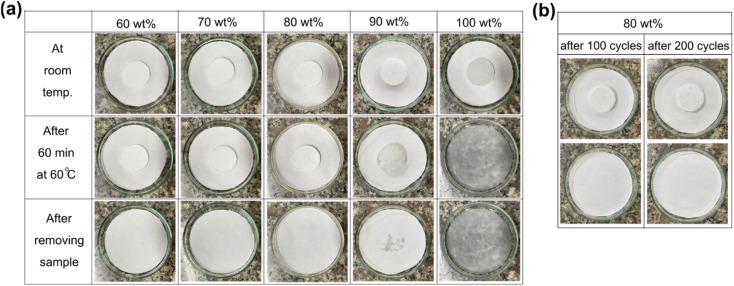
(a) Digital photos of PEG and the prepared SSPCMs during leakage test and (b) digital photos of the prepared 80 wt% PEG/FS SSPCM after 200 melting/crystallization cycles.

Shape-stability represents an SSPCM's capability to maintain its macroscopic shape and resist liquid leakage at temperatures above the melting point of PCM. Fig. 7 presents the shape-stability of the prepared PEG/FS SSPCMs compared to pure PEG after an isothermal treatment at 60 °C (∼17 °C above the melting point of PEG). The pure PEG was totally deformed due to melting, indicating poor shape-stability. Interestingly, the 60–90 wt% PEG/FS composites could maintain their original shape. However, the 90 wt% PEG/FS composite slightly leaked on its filter paper, attributed to some excessive PEG on FS surfaces (Fig. 2e). The 60–80 wt% PEG/FS composites showed excellent shape-stability without any leakage. As confined in FS porous network, melted PEG was stabilized by capillary, surface tension force, and interfacial H-bond interactions, effectively preventing liquid leakage.^17,19^ A higher content of PCM benefits the thermal performance of SSPCM since the thermal energy storage capacity is proportional to the amount of PCM. Therefore, the PEG/FS SSCPM with 80 wt% PEG content was selected as the optimal material. In addition, the shape-stability of 80 wt% PEG/FS SSPCM was further tested for 200 melting/crystallization cycles. As seen in Fig. 7b, the PEG/FS SSPCM could maintain the original shape without any leakage, indicating good shape stability and leakage resistance during multiple heat storage and release operations.

Table 2 compares the 80 wt% PEG/FS SSPCM to other SSPCMs from the literature in terms of optimal PCM content and thermal energy storage capacity. Overall, the prepared 80 wt% PEG/FS SSPCM exhibited comparable or even better PCM content and thermal energy storage capacity than the others and could be prepared at a low cost. On the one hand, when FS was used to support other PCMs such as *n*-octadecane, 1-octadecanol, and stearic acid, the optimal PCM contents were 70–75 wt%, lower than the value of 80 wt% of PEG. Compared to these PCM molecules, the PEG molecule has much more O atoms and –OH groups to form stronger interfacial H-bond interactions with silanol groups on FS surfaces.^18,19^ Consequently, a higher content of PEG could be stabilized in FS. On the other hand, PEG stabilized in other porous supports such as rice husk ash, silica hydroxyl, SBA-15 silica, mica, and diatomite presented lower thermal energy storage capacities and optimal PCM contents than that in FS. The PEG/orange peel-based carbon@nano Ag showed slightly better performance than the PEG/FS, however, its preparation required expensive reagents and complicated reaction processes.

**Table 2:** Comparison of the prepared 80 wt% PEG/FS SSPCM and others from the literature

SSPCM	Optimal PCM content (%)	Δ*H*_M_ (J g^−1^)	Ref.
*n*-Octadecane/FS	70	155.8	31
1-Octadecanol/FS	75	160.3	21
Stearic acid/FS	70	146.3	22
PEG/orange peel-based carbon@nano Ag	81.4	140.3	33
PEG/rice husk ash	62.1	119.3	16
PEG/silica hydroxyl	70.0	105.3	26
PEG/SBA-15 silica	70.0	0	18
PEG/mica	46.2	77.75	34
PEG/diatomite	71.5	121.5	35
PEG/FS	80	130.6	This work

### 3.4 Thermal conductivity of PEG/FS SSPCMs

**Fig. 8:**
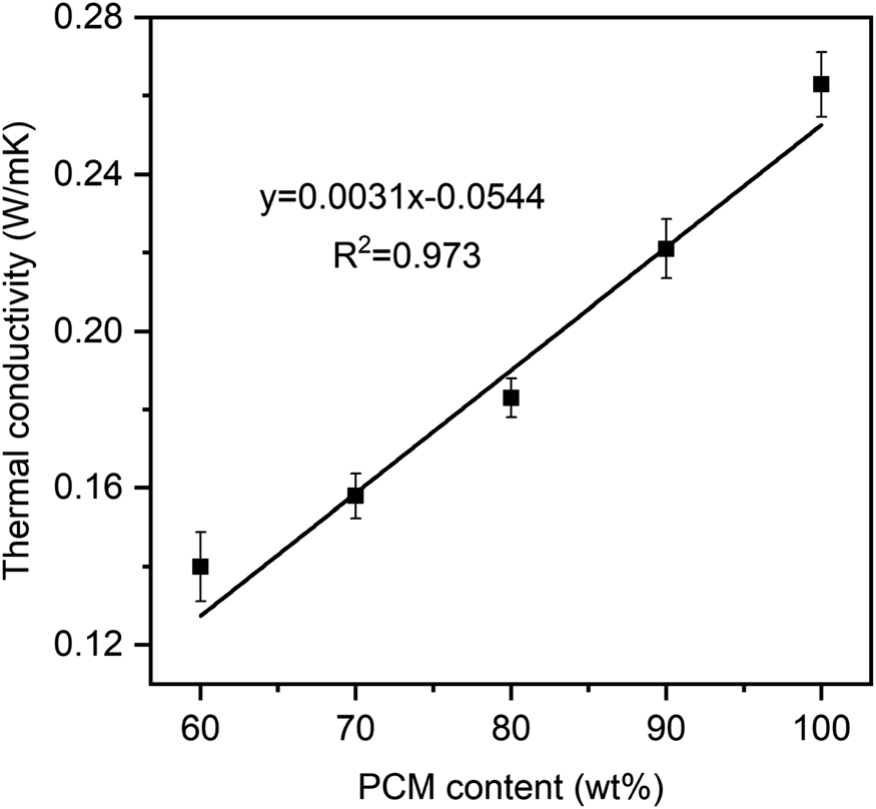
Thermal conductivity of pure PEG and the prepared PEG/FS SSPCMs.

Fig. 8 presents the linear correlation between thermal conductivity and the PEG ratio. Pure PEG had a low thermal conductivity of 0.263 W m^−1^ K^−1^, which was in good agreement with values of 0.242 and 0.2651 W m^−1^ K^−1^ from the literature.^16,33^ Meanwhile, the prepared PEG/FS SSPCMs with 60–100 wt% PEG presented increasing thermal conductivities of 0.140, 0.158, 0.183, and 0.221 W m^−1^ K^−1^, respectively. The thermal conductivity (*y*) and PEG content (*x*) could be correlated to a linear equation of *y* = 0.0031*x* − 0.0544, suggesting a steady growth in thermal conductivity with increasing PEG content. FS was known to possess a low thermal conductivity of ∼0.045 W m^−1^ K^−1^ because of the decreased gaseous thermal conductivity as the pore width was close to the mean path of free air (70 nm).^36^ When PEG was increasingly infiltrated into FS, the mesopores (Fig. 3b) and macropores (Fig. 2b–f) of FS were gradually filled. As a result, the thermal conductivity of the prepared PEG/FS composites gradually grew and the acquired values were between those of the two pristine substances. The low thermal conductivity of the prepared PEG/FS SSPCMs promoted heat transportation delay, making them appropriate for thermal insulation and thermal protection applications.

### 3.5 Thermal reliability of PEG/FS SSPCMs

**Fig. 9:**
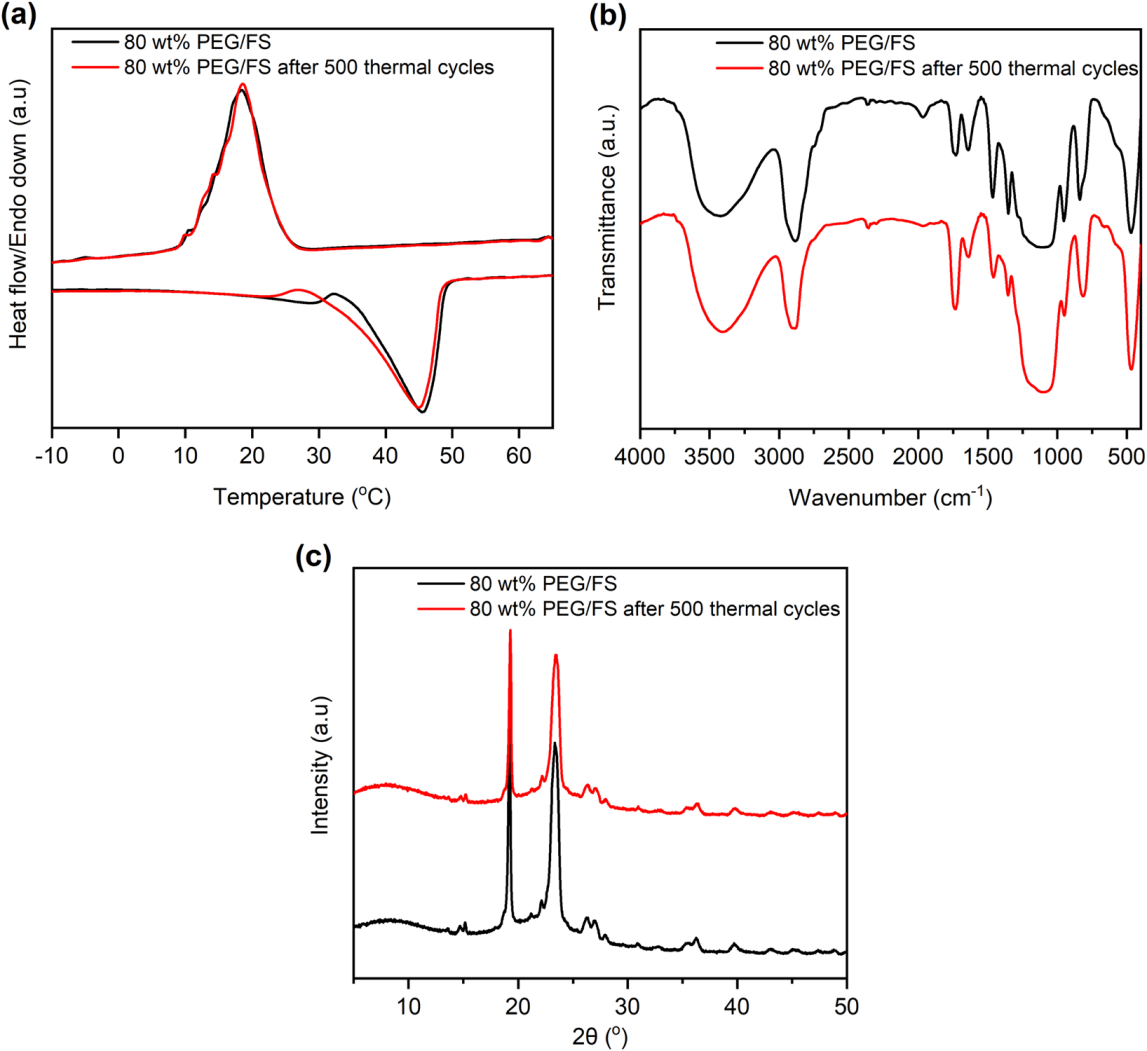
(a) DSC curves, (b) FTIR spectra, and (c) XRD patterns of the 80 wt% PEG/FS SSPCM before and after 500 melting/crystallization cycles.

Thermal reliability denotes the thermal durability of material after cyclic melting/crystallization operations and is a vital indicator for practical use. This work tested the thermal reliability of the optimal 80 wt% PEG/FS SSPCM with 500 melting/crystallization cycles. Fig. 9 shows the thermal reliability of the 80 wt% PEG/FS SSPCM characterized by DSC, FTIR, and XRD methods, and the detailed thermal properties are presented in Table 1. After the test, the sample presented a slightly different DCS curve compared to the original one (Fig. 9a). Specifically, the melting temperature decreased by 4.2 °C while the crystallization temperature was almost unchanged. More interestingly, the Δ*H*_M_ and Δ*H*_C_ were negligibly changed compared to those of the pristine cycle even after experiencing the repeated heat charge/discharge processes (see Table 1). The chemical stability of the 80 wt% PEG/FS SSPCM after the thermal cycles was further evaluated by FTIR spectra, as shown in Fig. 9b. No obvious difference was observed in absorbed peak positions and intensities of the samples before and after the test. In addition, the XRD patterns of the 80 wt% PEG/FS SSPCM after the thermal cycles (Fig. 9c) showed full characteristic peaks without change in relative intensity and diffraction angle, indicating the crystallization was maintained. These results demonstrated good thermal reliability, chemical stability, and crystallization for the prepared 80 wt% PEG/FS SSPCM after the multiple thermal cycles, highly qualified for long-term practical applications.

### 3.6 Thermal transfer retardation of PEG/FS SSPCM in building materials

**Fig. 10:**
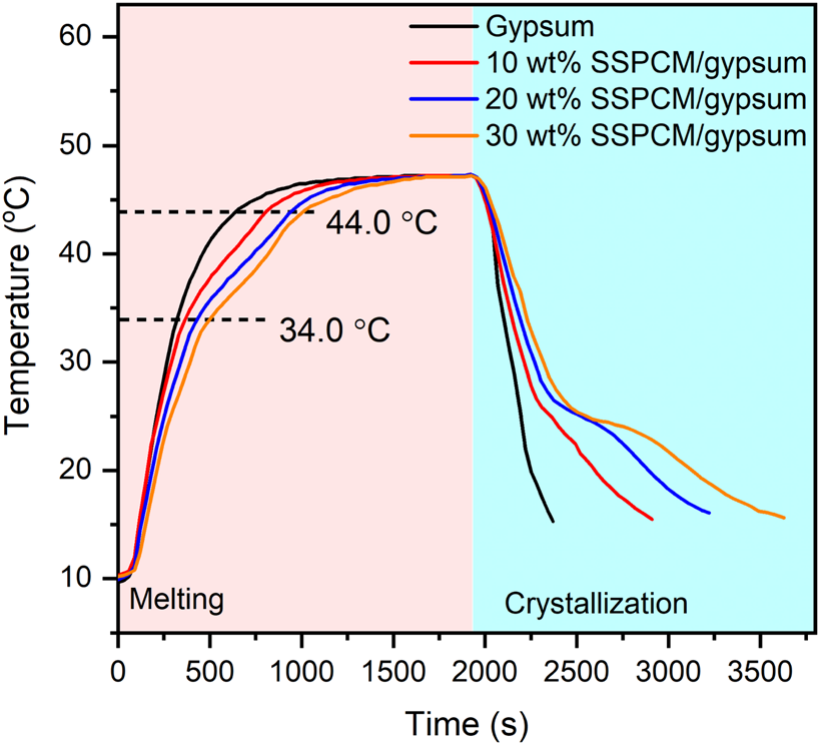
Temperature variation curves of gypsum and mixtures of gypsum and 80 wt% PEG/FS SSPCM with 10, 20, and 30 wt% SSPCM.

Fig. 10 compares the temperature variation of gypsum and gypsum combined with 80 wt% PEG/FS SSPCM (gypsum@SSPCM) at three different SSPCM contents of 10, 20, and 30 wt%. It can be explicitly seen that the gypsum@SSPCMs retarded the temperature growth compared to the neat gypsum, demonstrating that the composites absorbed a greater heat owing to the high latent heat storage capacity of the SSPCM. The temperature variation curves of the gypsum@SSPCMs occurred at three different phases determined by the tangential method. The first phase of <34.0 °C and the last phase of >44 °C presented a temperature growth before and after the melting of PEG/FS SSPCM governed by sensible heat storage. The middle phase (34–44 °C) exhibited the SSPCM's melting governed by both latent heat and sensible heat storage. Therefore, a slower temperature increase was achieved for the middle phase in comparison with the others. In contrast, the neat gypsum presented a fast temperature growth because of an absence of latent heat absorption. For comparison, to reach 44.0 °C, the neat gypsum needed only 660 s, while the gypsum incorporated with 10, 20, and 30 wt% SSPCM required 840, 960, and 1020 s, respectively. These results indicated that the gypsum@SSPCMs could narrow the temperature variation, highly applicable for energy-saving buildings.

## 4. Conclusions

A series of PEG/FS SSPCMs with increasing PEG content has been prepared by a simple solvent-assisted impregnation method, and the effects of confined PEG on thermal behaviors were studied. FS possessed an interconnected porous network with a dominant proportion of macropores and a minor amount of micro–mesopores. PEG was infiltrated into FS porous network by first filling the micro–mesopores, and then macropores. The interfacial H-bond interactions limited the crystallinity of confined PEG and reduced thermal energy storage capacity. Increasing PEG content in SSPCM increased the crystallinity because the PEG ratio in contact with FS surfaces was reduced. The 80 wt% PEG/FS SSPCM exhibited excellent thermal reliability after 500 accelerated thermal cycles, high crystallinity (93.1%) and thermal energy storage capacity (130.6 J g^−1^), and a low thermal conductivity (0.183 W m^−1^ K^−1^), which allowed for narrowing the temperature variation of SSPCM incorporated-gypsum as building materials. With the abovementioned merits and low cost, the PEG/FS SSPCMs have potential for large-scale utilization in energy-saving buildings.

## Conflicts of interest

There are no conflicts to declare.

## Supplementary Material
